# Neurohumoral Activation in Heart Failure

**DOI:** 10.3390/ijms242015472

**Published:** 2023-10-23

**Authors:** Antonis A. Manolis, Theodora A. Manolis, Antonis S. Manolis

**Affiliations:** 1 First Department of Cardiology, Evagelismos Hospital, 106 76 Athens, Greece; a.a.manolis@gmail.com; 2 Department of Psychiatry, Aiginiteio University Hospital, 115 28 Athens, Greece; doramanol9@gmail.com; 3 First Department of Cardiology, Ippokrateio University Hospital, 115 27 Athens, Greece

**Keywords:** neurohumoral activation, neurohormonal activity, beta blocker, angiotensin-converting enzyme inhibitor, angiotensin receptor blocker, neprilysin inhibitors

## Abstract

In patients with heart failure (HF), the neuroendocrine systems of the sympathetic nervous system (SNS), the renin–angiotensin–aldosterone system (RAAS) and the arginine vasopressin (AVP) system, are activated to various degrees producing often-observed tachycardia and concomitant increased systemic vascular resistance. Furthermore, sustained neurohormonal activation plays a key role in the progression of HF and may be responsible for the pathogenetic mechanisms leading to the perpetuation of the pathophysiology and worsening of the HF signs and symptoms. There are biomarkers of activation of these neurohormonal pathways, such as the natriuretic peptides, catecholamine levels and neprilysin and various newer ones, which may be employed to better understand the mechanisms of HF drugs and also aid in defining the subgroups of patients who might benefit from specific therapies, irrespective of the degree of left ventricular dysfunction. These therapies are directed against these neurohumoral systems (neurohumoral antagonists) and classically comprise beta blockers, angiotensin-converting enzyme (ACE) inhibitors/angiotensin receptor blockers and vaptans. Recently, the RAAS blockade has been refined by the introduction of the angiotensin receptor–neprilysin inhibitor (ARNI) sacubitril/valsartan, which combines the RAAS inhibition and neprilysin blocking, enhancing the actions of natriuretic peptides. All these issues relating to the neurohumoral activation in HF are herein reviewed, and the underlying mechanisms are pictorially illustrated.

## 1. Introduction

**Figure 1:**
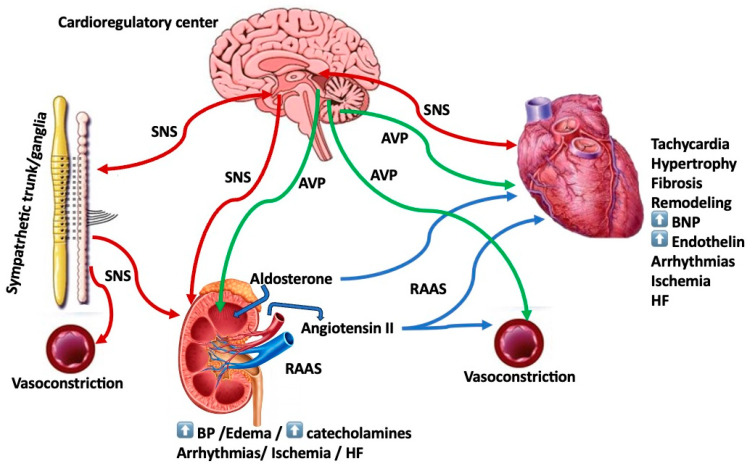
This is the schema of the intricate relationship between the various aspects and biological systems of neurohumoral activation and components of the heart failure (HF) syndrome (see text for discussion). AVP = arginine vasopressin; BNP = brain natriuretic peptide; BP = blood pressure; HF = heart failure; RAAS = renin–angiotensin–aldosterone system; SNS = sympathetic nervous system.

In patients with heart failure (HF), various neuroendocrine systems may be activated and produce the often-noted tachycardia and concomitant increased systemic vascular resistance [1,2]. Furthermore, sustained neurohormonal activation plays a key role in the progression of HF [3]. Among these systems, the renin–angiotensin–aldosterone system (RAAS), the arginine vasopressin (AVP) system and the sympathetic nervous system (SNS) may be responsible for the pathogenetic mechanisms leading to the perpetuation of the pathophysiology and worsening of the HF signs and symptoms (Figure 1, Table 1). Furthermore, signaling pathways of these neurohormonal systems, including the beta adrenergic and RAAS pathways, have been shown to be responsible for maladaptive remodeling processes and arrhythmogenicity [4]. Importantly, these systems may also constitute targets for HF therapies [5,6]. Interestingly, neuroendocrine activation starts as early as left ventricular (LV) dysfunction develops even before the emergence of HF, further heightened with the development of clinical HF [7]. With regard to HF therapy, neurohumoral-directed approaches, such as beta blockers, RAAS and angiotensin-converting enzyme (ACE) inhibitors and/or mineralocorticoid receptor antagonists (MRAs), and more recently, AVP antagonists seem to be a more logical approach with proven benefits. More recently, this neurohormonal approach has encompassed angiotensin receptor–neprilysin inhibitors (ARNi) (sacubitril/valsartan) and sodium-glucose co-transporter-2 (SGLT2) inhibitors (gliflozins) (see text for discussion) [6,8,9].

## 2. Sympathetic Nervous System (SNS)

Among the three neurohumoral systems, more consistently one observes the elevation of catecholamines and SNS activation that are responsible for the excessive vasoconstriction, the persistent tachycardia and attendant cardiac arrhythmias [10] (Figure 1). The excess of catecholamines results predominantly from increased SNS activity in the heart and kidneys while it coincides with decreased myocardial catecholamine level and correlates with a compromised left ventricular (LV) function, specifically reduced stroke volume and ventricular preload and portends a poor prognosis. A plasma norepinephrine level of >600 pg/mL may indeed be an indicator of compromised survival and grim prognosis [11].

Studies propose that increased SNS neurostimulation with resultant elevated plasma catecholamines may account for a reduction in the density of the beta adrenergic receptors or downregulation of these receptors, within the failing myocardium [12]. This may result in markedly attenuated responsiveness to inotropic and chronotropic stimuli [13,14].

An ostensible clinical picture that reflects the myocardial toxicity of excess of catecholamines, albeit reversible, is best illustrated in the case of pheochromocytoma [15]. Similar harm is expected and indeed encountered, perhaps to a lesser degree but nevertheless commensurate with the degree and duration of hypercatecholaminemia, in patients with HF. An important caveat concerning patients with HF relates to the desensitization of beta receptors that may develop in these patients as a result of sustained higher levels of norepinephrine in this group. The resultant downregulation with the reduction in the number of available receptors deprives the failing heart of an important inotropic stimulation. Furthermore, prolonged cardiac SNS hyperactivity can lead to deprivation of norepinephrine stores in the heart, and in such a scenario, the failing myocardium depends exclusively on circulating catecholamines to preserve sufficient cardiac output [14]. This results in a vicious cycle where the compensatory merit of inotropic support is compromised by the indiscriminate effect of catecholamines on circulation with a resultant increase in vascular resistance mediated via the activation of alpha adrenergic receptors, thus leading to increased afterload, compromised cardiac performance and worsening of HF [14,16].

Another important caveat in HF patients with heightened sympathetic activity concerns the development of *chronotropic incompetence* (CI), exercise intolerance and compromised exercise capacity in these patients that are associated with both central and peripheral autonomic derangements [17]. CI is the inability to sufficiently raise the heart rate from rest to exercise as correlated with the activation of the SNS and a consequent increase in circulating norepinephrine (NE). The downregulation of β-adrenergic receptors, usage of β-blockers, increased baseline heart rate or a combination of factors may account for this CI. Indeed, high NE levels alter chronotropic responses in HF patients and as a consequence result in compromised exercise capacity. A potential treatment of CI may be considered with the use of rate-adaptive pacing, applying a sensor for physical activity adjustment, without causing harmful neurohormonal activation of the SNS.

Yet another prominent manifestation of catecholamine toxicity relates to the development of the *Takotsubo syndrome (TTS)* or *stress cardiomyopathy or apical ballooning* [18,19,20]. Takotsubo syndrome is often triggered by intense emotional or physical stress; it occurs most commonly (~90%) in post-menopausal women, manifesting as an acute coronary syndrome (ACS), similar to an ST elevation myocardial infarction (STEMI); it presents as an acute HF syndrome or antero-apical cardiomyopathy with reversible left ventricular (LV) dysfunction involving LV walls in more than one coronary territory, usually in a global pattern, and commonly, albeit not exclusively, without significant underlying coronary artery lesion(s) [19,21]. The annual incidence of TTS is ~1–2% in patients undergoing coronary angiography for suspected ACS; the prognosis of this syndrome remains poor, with mortality comparable to acute MI (MI mimic) [19,21,22]. A major mechanism for the development of TTS relates to an acute cardiac sympathetic surge with norepinephrine release and spillover; however, circulating catecholamines may not have a direct effect in the pathogenesis of TTS, as the sympathetic activation and surge in norepinephrine release is confined to sympathetic nerve terminals and is considered to play a critical role in the acute phase of TTS producing acute myocardial stunning or paralysis [23,24]. Its prognosis is not as benign as one may think, as it may cause mechanical complications (cardiac rupture) and potentially lethal arrhythmias and sudden cardiac death (SCD) [18,22].

**Beta Blockers**. In the animal model, denervation-induced depletion of catecholamines causes an elevation in the density of beta adrenergic receptors and restoration of physiologic responsiveness [25]. In analogy and in keeping with these findings, an increased number of beta adrenergic receptors was also shown in human and animal studies following the administration of certain beta blockers; this may result not only in the recovery of adrenergic receptor sensitivity to agonist activity but also in ameliorated survival in patients with congestive cardiomyopathy [26,27,28].

The question whether beta blockers may furnish extra advantage in patients already taking aldosterone antagonists was addressed in the COPERNICUS trial of 2289 patients with severe HF treated with the beta1-beta2/alpha1 blocker carvedilol compared with placebo [29,30]. A post hoc analysis of two groups (445 patients receiving spironolactone and 1844 patients not on the drug at baseline) indicated that the beneficial action of carvedilol was the same among patients who were or were not taking spironolactone for each of the four efficacy indices [29]. For all-cause death rate, the Cox model HR for carvedilol vs. placebo was 0.65 in patients taking spironolactone and 0.65 in patients not on spironolactone; HRs for death or all-cause hospitalization were 0.76 vs. 0.76; for death or CV hospitalization, 0.61 vs. 0.75; and for death or HF hospitalization, 0.63 vs. 0.70, in patients taking and not taking spironolactone, respectively. The safety and tolerability of carvedilol treatment were also similar, irrespective of background spironolactone. The authors concluded that carvedilol remained clinically efficacious in the COPERNICUS trial of patients with severe HF when additionally administered to background spironolactone in patients who were virtually all being treated with an angiotensin-converting enzyme inhibitor (or angiotensin II antagonist). Thus, the use of spironolactone in patients with severe HF does not exclude the necessity of added anti-SNS therapy.

**Limitations/Practical Issues**. One limitation of beta blocking therapy in HF relates to the mode of initiation of such therapy, as one needs to start with low dosing in order to avoid HF worsening and acute decompensation [31].

Another limitation relates to chronotropic incompetence (CI), the inability to adequately raise the heart rate (HR) from rest to exercise, frequently noted in HF patients, especially when receiving beta blocker therapy. CI may occur because of the downregulation of β-adrenergic receptors, β-blocker use, high baseline HR or a combination of such etiologies. Elevated norepinephrine levels in these patients contribute to altered chronotropic responses in HF patients and consequently result in compromised exercise capacity. One potential countermeasure to deal with CI in this group of patients could be rate-adaptive pacing, employing a sensor to track physical activity, without causing neurohormonal activation of the sympathetic system with its attendant consequences [17].

## 3. Arginine Vasopressin (AVP)

Plasma arginine vasopressin (AVP) concentrations are high in patients with congestive HF and may participate in the disease progression via excess signaling at the V1a or V2 receptors [32,33,34]. Increased AVP signaling at either or both the V1a and V2 receptors may be implicated in the pathophysiology of HF by various mechanisms. V1a activation may lead to vasoconstriction with attendant increased systemic vascular resistance and afterload and/or direct myocardial hypertrophy. In addition, coronary vasoconstriction can occur resulting in reduced coronary blood flow and cardiac contractility [33]. As intracellular signaling pathways are closely linked to those for angiotensin II, V2 activation could lead to fluid retention, edema and hyponatremia.

There are three receptor subtypes that effectuate the actions of AVP (V1-A, V1-B and V2) [35]. The activation of V(1A) receptors found in vascular smooth muscle cells and the heart produces vasoconstriction and augmented afterload and hypertrophy. The V(2) receptors found mainly in the collecting tubules facilitate free water absorption. The V(1B) receptors are situated in the anterior pituitary and facilitate the release of adrenocorticotropin hormone. The CV and renal effects of AVP are brought about mainly by V1-A and V2 receptors. The antagonism of V1-A receptors produces vasodilatation, and the antagonism of V2 receptors results in aquaresis, an electrolyte-sparing water excretion.

**Vasopressin Antagonists/Vaptans.** Many non-peptide AVP antagonists or “vaptans” have been produced and are being evaluated mainly for managing hyponatremia and fluid overload. *Conivaptan* is a combined V1-A/V2-receptor antagonist that induces diuresis and leads to hemodynamic improvement. In clinical trials, this agent has been found to restore euvolemic and hypervolemic hyponatremia and has been granted approval by the US FDA for the management of euvolemic hyponatremia as an intravenous infusion. *Tolvaptan*, a selective V2-receptor antagonist, has been successful as tested clinically in treating hyponatremia in volume overloaded patients with HF. The results are pending of a large CV outcome trial (n = 4133 patients) assessing its role in the treatment of HF. Lixivaptan and satavaptan (SR-121463) are other selective V2-receptor antagonists being assessed for the management of hyponatremia.

Tolvaptan, conivaptan, lixivaptan and satavaptan are some of the vasopressin antagonists that have been tried in HF patients. The outcomes were initially promising with the relief of symptoms and adequate aquaresis without aggravating hyponatremia or kidney function; however, they did not succeed to demonstrate any influence on the death rate in HF [36]. With the availability of more selective orally active vasopressin antagonists, additional trials have been performed in HF patients and other disease states with volume overload and hyponatremia [35].

A hemodynamic study with the pure V2 antagonist tolvaptan indicated minimal hemodynamic influences [37]. In another study comprising 14 patients with mild to moderate HF, the renal and neurohormonal influences of tolvaptan were favorable in comparison with furosemide; both tolvaptan and furosemide raised urine volume, but tolvaptan preserved renal blood flow which was reduced by furosemide without influencing urine or serum sodium levels; there was no change in neurohormone levels in the tolvaptan group while furosemide raised renin and norepinephrine concentrations [38].

Several clinical studies with tolvaptan as add-on treatment in acute HF have demonstrated advantageous effects on fluid balance and shortness of breath, with no deterioration in renal function or neurohormonal activation [32]. Similar clinical and more favorable renal and neurohormonal actions of tolvaptan versus loop diuretics were shown in two smaller trials, one in acute and one in chronic HF. However, long-term therapy with tolvaptan did not affect outcomes in acute HF. Scarce data are available limited to single-dose trials of an intravenous pure V1-A antagonist, which disclosed a vasodilating effect if plasma AVP concentrations were high. Hemodynamic and clinical effects largely similar to those with tolvaptan in similar trials were shown by one hemodynamic trial and one short-duration clinical study with the balanced intravenous V1-A/V2 antagonist conivaptan. A new oral agent, pecavaptan, an efficacious balanced V1/V2 antagonist, is currently tested in a phase II trial as both adjunctive and alternative treatment during and after hospitalization for acute HF [32].

Importantly, vaptans, which promote aquapheresis, appear useful in treating chronic nonhypovolemic hypotonic hyponatremia, as all four available vaptans (lixivaptan, satavaptan, tolvaptan and satavaptan) have been demonstrated to improve serum sodium concentration without altering renal hemodynamics [39,40].

With regard to the impact of vaptans on the death rate in HF patients, some studies were not powered to assess mortality, but a post hoc analysis of a study with tolvaptan (ACTIV in CHF) indicated a decrease in a short-term death rate [41]. In keeping with this finding, a post hoc analysis of another study (EVEREST) also showed better survival in patients with serum sodium <130 meq/L in addition to improvement in dyspnea conferred by tolvaptan [42]. The main study did not show an effect on mortality despite the obvious short-term clinical benefits [43].

Finally, a meta-analysis of eight RCTs examined the efficacy and safety of tolvaptan in treating HF in 13,453 hospitalized patients [44]. There was a considerable decrease in body weight (mean difference, MD = −0.87; *p* < 0.001) among all subgroups. Serum sodium was significantly increased by tolvaptan at day 1 (MD = 2.93, *p* < 0.001) and at day 7 or discharge (MD = 3.10, *p* < 0.001). Dyspnea (RR = 1.10, *p* < 0.001) and edema (RR = 1.05, *p* < 0.001) improved significantly, whereas there was no difference in rales and pulmonary congestion. There were no important adverse events.

## 4. Renin–Angiotensin–Aldosterone System (RAAS)

Renin catalyzes the conversion of a plasma protein, angiotensinogen, which is produced in the liver, to create angiotensin I, a peptide with only mild vasoconstrictor effects [45]. The angiotensin-converting enzyme (ACE) is a nonspecific enzyme, found primarily in the endothelium of lung vessels, catalyzing the conversion of angiotensin I into angiotensin II [45]. Other tissues, like the kidneys and blood vessels, have the enzyme, allowing local formation of angiotensin II. Angiotensin II is a robust vasoconstrictor agent crucial in preserving the homeostasis of the circulation; it is often swiftly inactivated by various angiotensinases. It binds to two receptors, angiotensin type I (AT 1) and angiotensin type 2 (AT 2), with opposing actions. In the heart muscle, the ratio of AT 2 to AT 1 is 2:1; however, the expression of AT 1 receptors increases in HF, producing vasoconstriction, secretion of aldosterone, cell growth and secretion of catecholamines. Thus, the RAAS plays a cardinal role in the mechanism of congestive HF. The overactivation of the RAAS leads to ardent salt/water retention and left ventricular (LV) hypertrophy in HF.

During its persistence in the tissues, angiotensin II raises blood pressure by two mechanisms that include vasoconstriction with an attendant rise in peripheral resistance and venous return and a concomitant diminution in the renal excretion of sodium and water, raising extracellular fluid volume, either directly and/or via the stimulation of aldosterone secretion from the adrenal glands [46]. In HF, the formation of angiotensin II is deleterious, leading to the production of fibrosis in the heart and kidneys [47]. Angiotensin II also worsens neurohormonal activation, as it instigates the release of norepinephrine and also the production of aldosterone in the adrenal cortex. The independent effects of aldosterone on extracellular volume control comprise its binding to the mineralocorticoid receptor in the distal tubule and enhancing sodium retention and potassium excretion. Furthermore, aldosterone leads to myocardial and vascular fibrosis, direct vascular injury, baroreceptor dysfunction and cancels the uptake of norepinephrine by the heart muscle [48].

## 5. Counterregulatory Systems

The RAAS pharmacologic interventions mainly comprise the ACE inhibitors, the angiotensin II-AT1 receptor blockers, the mineralocorticoid receptor antagonists and the direct renin inhibitors [49]. Furthermore, the angiotensin receptor–neprilysin inhibitor (ARNI) sacubitril/valsartan is a more recent addition to enhance this counteraction of the RAAS activation [6]. In addition, neurohormonal systems that are active in HF patients to balance the actions of the vasoconstricting neurohormones comprise *natriuretic peptides (NPs)* (Table 1) released in response to increased atrial and myocardial stretch, unloading the heart by enhancing the renal excretion of salt and water and blocking the release of renin and aldosterone [50]. Besides diuresis and natriuresis, natriuretic peptides promote vasodilation by augmenting cyclic guanosine monophosphate-mediated smooth muscle relaxation and facilitating capillary permeability [51]. Despite great elevations in circulating natriuretic peptides, as HF advances, their renal action becomes progressively obtunded, permitting the RAAS influences to remain [51]. Natriuretic peptides are widely produced in several tissues and broken down mostly by internalization, followed by lysosomal and enzymatic degradation by neutral endopeptidase neprilysin [50]. The autonomic nervous system (ANS) and other local autoregulatory mechanisms interact to maintain blood flow in the brain and the myocardium while producing intense vasoconstriction to lower blood flow to other organs during exercise or injury. This process is facilitated by vasoconstricting neurohormones, which then activate counterregulatory vasodilator pathways to annul their detrimental influences. Bradykinin is one of these vasodilators secreted at the sites of inflammation and coagulation. It produces strong vasodilation mainly of the arteriolar and capillary bed. The enzymes ACE and neprilysin facilitate the degradation of bradykinin as well as the production of the strong vasoconstrictor angiotensin II [52].

## 6. Cardiac Remodeling

Cardiac remodeling is defined as a cluster of genetic, molecular, cellular and interstitial alterations that are set in place after cardiac load or injury, which results in increased heart volume and changes from elliptical to a spherical shape, leading to LV systolic and diastolic dysfunction [53]. In patients with HF, myocardial remodeling is a determinant of the clinical course of HF and confers a poor prognosis; conversely, when reversed it ameliorates CV outcomes [53,54,55]. Thus, it is important to forego the hemodynamic burden and the neurohormonal activation that lead to cardiac remodeling and LV dysfunction. Timely HF therapy might avert the development of cardiac remodeling and delay the progression of HF [56]. The all-cause death, CV death and all components of the CV death were shown to decline with increasing LV ejection fraction (LVEF) until an LVEF of ~45%, after which the risk of these CV outcomes relatively stabilized with increasing LVEF [54]. Reverse remodeling indicated by reduction in LV end–systolic volume conferred by cardiac resynchronization therapy (CRT) [57] is a powerful predictor of long-term survival in mild HF [55]. Indeed, CRT has been shown to ameliorate cardiac sympathetic nerve activity in responders and thus lead to reverse remodeling [58]. Also, long-term CRT was shown to be linked with reduced RAS activation and stabilization of NT-proBNP in HF patients with reverse remodeling [59].

## 7. Countering RAAS

Current guidelines recommend agents targeting this system, specifically in patients with HF with reduced ejection fraction (HFrEF), to extend survival and stabilize or reverse cardiac remodeling [60,61]. These agents comprise angiotensin-converting enzyme (ACE) inhibitors (ACEi), angiotensin receptor blockers (ARBs) and the angiotensin receptor–neprilysin inhibitor (ARNI) sacubitril/valsartan [60].

**ACE Inhibitors vs. Angiotensin Receptor Blockers**. Some investigators have suggested an advantage of ACEi vs. ARBs in treating HF, as in their meta-analysis, the data indicated that ACEi, but not ARBs, lowered the all-cause death rate and CV deaths [62]. Thus, they recommended that ACEIs should be considered as first-line therapy to curtail excess mortality and morbidity in this population. Specifically, the investigators analyzed the results of 38 studies assessing the use of these agents in 47,662 subjects who were followed up for a duration ranging from 12 weeks to 4.5 years [62]. Of all thirty-eight trials, thirty-two compared ACEi with baseline therapy (thirteen arms compared ACEi with placebo, in ten arms, the comparator was active therapy, and nine arms compared ACEi with ARBs), and six studies compared ARBs with placebo. Treating patients with HF with an ACEi lowered the all-cause death rate to 11% (risk ratio, RR: 0.89; 95% confidence interval (CI): 0.83–0.96; *p* = 0.001) and the respective value for the CV death rate was 14% (RR: 0.86; 95% CI: 0.78–0.94; *p* = 0.001). Interestingly, ARBs had no advantage in decreasing all-cause and CV death rate. However, in a head-to-head analysis, treatment with ACEIs was not superior to ARBs for all-cause and CV deaths.

While the adverse effects of angiotensin II (Ang II) in HF are well established, the biological actions of angiotensin 1–7 (Ang 1–7) are less clear. Chronic administration of Ang 1–7 or its agonist AVE 0991 exerted important diuretic, natriuretic and kaliuretic effects in HF rats, but not in sham controls [63]. Serum creatinine and aldosterone concentrations were much higher in vehicle-treated HF rats vs. controls. Treatment with Ang 1–7 and AVE 0991 decreased these measurements to levels similar to those noted in sham controls. Also, chronic administration of Ang 1–7 to HF rats decreases myocardial hypertrophy. The authors concluded that Ang 1–7 exerts beneficial renal and cardiac influences in rats with HF leading to the postulation that the ACE2/Ang 1–7 axis represents a compensatory reaction to the overactivity of ACE/AngII/AT1R system characterizing HF and indicating that Ang 1–7 may be a possible therapeutic modality in this situation. ACE2 has been considered a crucial member of the RAAS, as it performs an important regulatory function in the CV system. ACE2 counterregulates the RAAS mainly by hydrolyzing the harmful AngII to the beneficial Ang (1–7). Increasing or activating ACE2 and/or Ang (1–7) may help to prevent and treat myocardial ischemia reperfusion injury [64]. Ang (1–7) also has antihypertrophic actions [65]. It can thus delay the progression of HF and treatment with different activators of the antihypertrophic Ang II receptors (AT2R and MasR) under chronic pathological stages may prove beneficial. Thus, it is proposed that efforts should aim to develop appropriate activators of both AT2R and MasR for the treatment of HF patients [65]. However, additional research into the specific processes behind the ACE2/Ang (1–7)/MasR axis in this setting is necessary.

**Aldosterone receptor blockade**. In patients with mild–moderate HF, spironolactone decreased neurohumoral activation (as reflected by BNP levels) and an indicator of collagen turnover (PIIINP) but worsened renal function and quality of life [66]. The benefit–risk ratio of aldosterone blockade in mild HF was not certain and needed clarification in a large RCT. Indeed, the EMPHASIS-HF (N = 2737) trial indicated that eplerenone significantly reduced major CV events vs. placebo in patients with mild symptoms of HFrEF (LVEF < 35%) besides recommended therapy with an acceptable safety profile, even in those already receiving high doses of standard background therapies [67]. As mentioned, the use of spironolactone in patients with severe HF seems to complement anti-SNS therapy [29]. Importantly, aldosterone receptor blockade or mineralocorticoid receptor antagonism with the use of spironolactone or eplerenone reduces morbidity and mortality in patients with HF, including the elderly subgroup; this beneficial effect is more pronounced in patients with HFrEF, albeit it remains homogenous across HFrEF and HFpEF [68]. The caveat to be watched for with these therapies in HF patients is the risk of hyperkalemia and/or worsening renal function, and thus there is a need for close monitoring of potassium levels and renal function [69]. Nevertheless, trial data have indicated that in HF patients receiving optimal therapy, worsening renal function and hyperkalemia were, indeed, more frequent when eplerenone was added, but their occurrence did not eliminate the survival benefit of eplerenone [70]. Finally, a post hoc analysis of the EMPHASIS-HF trial indicated that eplerenone improves survival and may prevent readmission when initiated soon after a hospitalization for HF or an acute coronary syndrome in patients with HFrEF and mild symptoms, while it remains safe [71].

**Angiotensin Receptor–Neprilysin Inhibitor (ARNI).** In recent years, the RAAS blockade has been refined by the introduction of the angiotensin receptor–neprilysin inhibitor (ARNI) sacubitril/valsartan, which combines the RAAS inhibition and neprilysin blocking, enhancing the actions of natriuretic peptides [72]. The PARADIGM-HF trial showed a significant benefit of sacubitril/valsartan over enalapril in mitigating CV mortality and HF hospitalizations rates for patients with HFrEF. Also, several RCTs and observational trials examined its role in various clinical scenarios and its efficacy has been fully acknowledged in the most recent HF European and USA guidelines. The influence of sacubitril/valsartan on major CV outcomes leads to a decrease in NT-proBNP concentrations and produces reverse cardiac remodeling and a decrease in mitral regurgitation. Supplementing HF therapy with the usage of ARNI in clinical practice can ensure maximal treatment efficacy [73]. Furthermore, ARNi therapy seems to mitigate the risk of arrhythmic events requiring appropriate shock therapy by an implantable defibrillator device in patients with HFrEF [74].

The introduction of ARNi has ushered in a new therapeutic era in managing HF patients with the intent to raise vasodilatory natriuretic peptides and avert counterregulatory activation of the RAAS. Indeed, ARNi treatment is very efficacious in lowering the risks of death and hospitalization for HF in patients with HF and New York Heart Association (NYHA) functional class II to III symptoms, but trials have failed to demonstrate any advantages with ARNi versus ACE inhibitors or ARBs in patients with advanced HFrEF or in patients after myocardial infarction with attendant LV dysfunction but without HF [72]. These studies bring about questions about whether the enzymatic catabolism of natriuretic peptides may not be a very efficacious and desirable solution in patients with advanced HF when there is downstream blunted response to natriuretic peptides or among post-MI patients in the absence of HF when increased availability of peptides may not be needed.

## 8. Heart Failure with Preserved Ejection Fraction

Although HF with preserved ejection fraction (HFpEF) is a heterogeneous syndrome involving the interaction between heart diseases, comorbidities and ageing, the activation of neurohormonal axes is also implicated in its pathophysiology, particularly the activation of the RAAS and the sympathetic nervous system, albeit to a lesser degree compared with HF with reduced ejection fraction (HFrEF) [75]. Thus, neurohormonal modulation may also be a therapeutic strategy for HFpEF. However, RCTs have failed to show a beneficial effect on prognosis from neurohormonal modulation treatments in HFpEF, with the exception of patients who have a left ventricular ejection fraction (LVEF) in the lower normal range, whereby the American guidelines propose that such treatments may be considered. Importantly, the guidelines emphasize the need to treat comorbidities in these patients, such as hypertension, diabetes, anemia, atrial fibrillation (AF), sleep disorders [61].

Nevertheless, as detailed below, recent data support the use of these agents in a wide range of HF, regardless of LVEF. Among 12,251 patients with HF with mildly reduced or preserved LVEF in the DELIVER and EMPEROR-Preserved trials, SGLT2 inhibitors lowered the composite endpoint of CV or first hospitalization for HF (HR 0.80) with consistent decreases in both components of CV death (HR 0.88) and first hospitalization for HF (HR 0.74) [76].

## 9. Neurohormonal Biomarkers

Biomarkers, such as markers of the activation of neurohormonal pathways, that include the natriuretic system and the sympathetic nervous system, may all be used to better understand the mechanisms of HF drugs and also aid in the definition of subgroups of patients who might take advantage from specific therapies, irrespective of LVEF [77]. Classical biomarkers include catecholamine levels, natriuretic peptides (NPs), endothelin-1 (ET-1) and neprilysin [78]. Novel biomarkers, like mid-regional pro-atrial natriuretic peptide (MR-proANP), mid-regional proadrenomedullin (MR-proADM), high-sensitivity troponins, growth differentiation factor (GDF)-15, soluble ST2 (sST2) and galectin-3, show potential in prognosis beyond that which is rendered by NPs and other classical biomarkers, but their role in the clinical arena is still partially defined and further trials are required [79,80,81]. Interestingly, a more recent cohort study indicated that in HF patients or those at the risk of HF, before first hospitalization, GDF-15 grants specific information and is highly predictive of hospitalization for HF or all-cause death rate, contributing to more precise risk assessment that can better guide clinical decision making [80]. In addition to NPs, galectin-3 has been suggested as an important biomarker to stratify HF patients [82].

Certain neurohormonal biomarkers (ET-1, norepinephrine, aldosterone, plasma renin activity) and cytokines (TNF-α, IL-6) could predict survival in patients hospitalized for acute HF. It has been shown that measuring ET-1 levels provides significant predictive information on the risk of death after hospital discharge [83,84]. Furthermore, ET-1 seems to have a higher discriminatory power than other neurohormones and cytokines for risk stratifying HF patients. Specifically, a study suggested that high endothelin-1 levels may be implicated in enhancing the emergence of ventricular ectopy in patients with decompensated HF; such proarrhythmic actions may be responsible, in part, for the poor outcome conferred by high endothelin-1 concentrations in these patients [84].

**Natriuretic Peptides**. Among the various pathophysiological mechanisms that influence the secretion of the brain natriuretic peptide (BNP) in patients with HF, which include neurohormonal activation, cardiac dysfunction, myocardial stretch and elevated filling pressure, neurohormonal activation remains cardinal [85,86]. This natriuretic peptide system establishes the heart as an endocrine organ [87]. The biological action of BNP tends to counterbalance the pathophysiological mechanisms that lead to the initiation and advancement of HF by enhancing natriuresis and diuresis and by blocking neurohormonal activation and cardiac remodeling [51].

Natriuretic peptides (NP), the brain natriuretic peptide (BNP) and N-Terminal-pro-BNP (NT-pro-BNP; a fragment of the BNP precursor) represent the most extensively employed biomarkers in HF patients; their role has been stablished in diagnosis and risk stratification in these patients [88,89]. They are a group of molecules secreted in response to myocardial stretch, with pertinent effects on natriuresis, vasodilatation and curtailing fibrosis, explored for their prognostic and therapeutic uses [90,91]. Circulating levels of the BNP and NT-pro-BNP parallel the NYHA functional class and have an independent linear relationship with in-hospital death in patients hospitalized for decompensated HF, either HF with reduced ejection fraction (HFrEF) or HF with preserved ejection fraction (HFpEF) [79,92]. Finally, NT-proBNP levels have been linked with the risk of incident ventricular arrhythmias after correcting for established risk factors, with the strongest link seen in patients who have an indication for an ICD for secondary prevention of SCD [93].

**Endothelin**. Among several biomarkers, endothelin-1 (ET-1) seems to be involved in many facets of the pathogenesis of acute HF including neurohormonal activation, endothelial dysfunction, cardiac remodeling, inflammation, atherosclerosis and altered renal function [83]. Since its discovery, a plethora of studies have indicated that the level of ET-1 is linked with the severity of symptoms and myocardial dysfunction in this pathology; other data indicate that increased endothelin-1 levels may predict the emergence of ventricular ectopy in patients with decompensated HF [83,84].

**Lymphocyte G-protein-coupled Receptor Kinase 2 (GRK2)**. GRK2 levels mirror myocardial β-adrenergic receptor function in HF and have been demonstrated to have additional independent prognostic information regarding ANS activation [88].

**Neprilysin**. Neprilysin is a transmembrane endopeptidase implicated in the breakdown of various vasoactive peptides and may serve as a therapeutic goal in HFrEF. According to a study investigating the relation of circulating neprilysin with neurohumoral activation and the influence of plasma neprilysin activity on prognosis in HFrEF in 369 chronic HFrEF patients, no correlation was found between plasma neprilysin levels and neurohumoral activity (*r*_s_ = 0.09, *p* = NS) [94]. Plasma neprilysin activity correlated with the severity of HF as determined by New York Heart Association stage (*p* = 0.003) and the tertiles of the N-terminal pro-B-type natriuretic peptide levels (*p* < 0.001), whereas neprilysin levels did not (*p* = 0.220; *p* = 0.849). There was no link between plasma neprilysin levels and neurohumoral activity, as determined by absolute renin level (*r*_s_ = −0.02, *p* = 0.648; *r*_s_ = 0.03, *p* = 0.574) or norepinephrine concentrations (*r*_s_ = −0.06, *p* = 0.248; *r*_s_ = 0.20, *p* < 0.001). Neither circulating neprilysin levels nor activity were linked with the outcome.

**Plasma Renin Activity (PRA).** The prognostic role of PRA as a specific neurohumoral biomarker of the RAAS and sympathetic activation pathways in HF was assessed in a population of 996 patients with HFrEF on optimal therapy treatment (LVEF < 50%, mean age 65 ± 13 years), who were followed up for a median 36 months (range 0 to 72) for CV death and appropriate implantable cardioverter device (ICD) shock [95]. A total of 170 CV deaths and 27 shocks were recorded. In a Cox multivariable analysis, only LVEF (hazard ratio, HR, 0.962), NT-proBNP (HR 1.729) and PRA (HR 1.201) were independent prognosticators of CV mortality. A receiver operating characteristic curve analysis revealed a cutoff value for PRA of 2.30 ng/mL/h that best presaged CV death. Independent prognosticators of PRA were LVEF, functional NYHA class, potassium, sodium, NT-proBNP, norepinephrine, aldosterone, C-reactive protein and medical treatment. The link of high NT-proBNP and high PRA defined a subset (22% of the study population) with the highest risk of CV death. The authors suggested that PRA might assist in the selection of those patients requiring an additional therapeutic attempt, possibly aiming at incomplete renin–angiotensin–aldosterone system blockade.

**Catestatin**. Catestatin is a multifunctional 21-aminoacid peptide involved in metabolic homeosta-sis and in the regulation of the CV and immune systems; it inhibits catecholamine se-cretion and thus mitigates the deleterious activity of the SNS [96]. Catestatin, a prod-uct of the precursor chromogranin A, is a new regulator of myocardial function and blood pressure, a powerful physiological inhibitor of catecholamine spillover that has cardioprotective actions [97,98]. High levels of catestatin appear to parallel SNS hy-peractivity and increased catecholamine secretion. It is helpful in risk stratification of patients with HF, while it may also be a prognosticator of malignant ventricular ar-rhythmias in patients with acute myocardial infarction (MI) [97].

In the peripheral system, catestatin has a hypotensive effect by stimulating histamine secretion from mast cells, blocking catecholamine release from the sympathoadrenal system and producing vasodilation [99]. Centrally, catestatin ameliorates baroreflex sensitivity and heart rate variability by various mechanisms involving γ-aminobutyric acid GABAergic neurons and glutamatergic rostral ventrolateral medulla neurons [99]. In addition, catestatin has a cardioprotective effect by inhibiting inotropy and lusitropy, activating mitochondrial KATP channels and enhancing reperfusion injury salvage kinase production and survivor-activating factor enhancement pathways and subsequent inhibition of mitochondrial permeability transition pore. Catestatin regulates cardiomyocyte Ca^++^ concentrations by direct inhibition of Ca^++^/calmodulin-dependent protein kinase IIδ activity and a consequent decrease in the phosphorylation of phospholamban and ryanodine receptor 2 actions which provide evidence for a direct functional role of catestatin in the failing heart [99].

## 10. Diuretics/Ultrafiltration

Heart failure is linked with enhanced sodium avidity and overload of extracellular volume, with its dire consequences of lung congestion and peripheral edema that may culminate into pulmonary edema or ascites and anasarca, respectively [100]; in such cases, management is directed toward diuretic use. However, diuretic efficacy in HF may be limited by adverse neurohormonal activation [101]. Such a mechanism (neurohumoral activation) may account for some cases of so-called diuretic resistance in HF patients [102]. Similarly, ultrafiltration, even though a promising method to obtain a more rapid fluid and weight loss in HF patients, particularly in those with large fluid retention (e.g., ascites), has also been shown to instigate neurohumoral activation, besides worsening renal function and hypotension, similar to those produced by loop diuretics [103].

## 11. SGLT2 Inhibitors

Sodium glucose cotransporter-2 (SGLT2) inhibitors or gliflozins improve HF-related outcomes and this beneficial influence occurs irrespective of the presence or absence of type 2 diabetes [9,104,105,106,107]. The mechanisms accounting for these benefits are not well understood, but drug-induced osmotic diuresis and natriuresis seem to be the main contributing mechanisms, together with the preservation of glomerular filtration and facilitation of interstitial drainage, which can collectively translate into effective and safe decongestion [108]. Traditional diuretics such as furosemide promote the excretion of Na^+^ and water by the kidneys by blocking their reabsorption from the proximal and distal tubules and from the loop of Henle; however, these agents lead to substantial neurohormonal activation, resulting in the limited improvement in intravascular volume often observed with these agents. On the other hand, the proximal tubular site of action of the SGLT2 inhibitors might bypass these limitations. Empagliflozin produces significant natriuresis, particularly when combined with loop diuretics, causing an improvement in blood volume. However, renal dysfunction, off-target electrolyte wasting and neurohormonal activation have not been encountered [109]. This advantageous diuretic profile may provide a significant benefit in the management of volume status in patients with HF and may constitute a mechanism leading to the superior long-term HF outcomes noted with these agents.

Thus, SGLT2 inhibitors could be viewed as neurohormonal antagonists when prescribed for the management of HFrEF [110]. Their benefits extend beyond glycemic control since the inhibition of renal glucose reabsorption has an effect on blood pressure and ameliorates the hemodynamic profile and the tubule glomerular feedback; such an effect can rebalance the dense macula response by restoring adenosine formation and limiting renin–angiotensin–aldosterone activation [111]. Furthermore, there is also evidence presented which supports the notion of potential sympatholysis exerted by SGLT2 inhibitors independently of their glucose-lowering and diuretic effects [112].

As already mentioned, recent data support the use of these agents in a wide range of HF, independent of LVEF. Among 12,251 persons participating in the DELIVER and EMPEROR-Preserved trials, SGLT2 inhibitors lowered the composite endpoint of CV or first hospitalization for HF (HR 0.80) with consistent decreases in both the components of CV mortality (HR 0.88) and the first hospitalization for HF (HR 0.74) [76]. In the wider context of the five studies of 21,947 participating individuals, the SGLT2 inhibitors mitigated the risk of composite CV mortality or hospitalization for HF (HR 0.77), CV mortality (HR 0.87), first hospitalization for HF (0.72) and all-cause mortality (HR 0.92). These treatment results were consistent for HF with mildly reduced or preserved LVEF and across all five studies. The authors concluded that SGLT2 inhibitors reduced the risk of CV mortality and HF hospitalizations in a wide range of HF patients, backing up their role as main HF therapy, regardless of LVEF or care setting.

Importantly, antiarrhythmic actions have been ascribed to SGLT2 inhibitors via various mechanisms including reverse atrial and ventricular remodeling, improved mitochondrial function, fewer hypoglycemic events with a consequent reduction in their proarrhythmic effects, curtailed SNS activity, better control of sodium and calcium homeostasis and shortening of lengthened ventricular repolarization [107].

## 12. Neurohumoral Blockade in Various Clinical Settings/Scenarios

**Neurohormonal Blockade in Older Patients with HF.** As detailed above, due to the sustained neurohumoral activation that occurs in HF patients in response to compromised (systolic) pumping and/or (diastolic) filling properties of the heart, the clinical utility of neurohormonal blockers has revolutionized the management of these patients over the past 30 years. This neurohormonal blockade has led to reduced morbidity and mortality in this population, particularly in those with HFrEF and sinus rhythm [113]. The survival benefit of this neurohumoral blockade strategy extends to all age groups, including the very old (≥80 years), whereby an average 40% mortality reduction has been reported [114]. A potential caveat in this particular group relates to the inability of older patients to tolerate the recommended doses of such therapy. In this regard, a study comprising 185 older (>80 years) patients with HF and LVEF ≤ 40%, indicated that only 53% tolerated and took the target dose of ACEIs/ARBs, whereas 26% had <50% of the target dose [115]. Half took <50% of the target dose of beta blockers and 21% had the target dose. After ≥5 years of follow-up, the all-cause death rate was 77%. Patients who took the target dose of ACEIs/ARBs had increased survival rates from all-cause death than those having <50% of target dose (hazard ratio, HR = 0.6; *p* = 0.033), but those having ≥50% of target dose had similar benefit with those who achieved target dose. The clinical outcome of beta blocker treatment was not dependent on the drug dose when the target heart rate could be attained.

Registry data from 48,711 patients (aged ≥65 years) hospitalized with HFrEF have been recently analyzed showing important links between beta blocker and/or RAS inhibitor usage at hospital discharge and decreased 30-day and 1-year death rates, including in those over the age of 85 (30-day hazard ratio, HR, 0.56; 1-year HR 0.69) [116]. Furthermore, the degree of benefit linked with the BB and/or RASi usage after discharge was not mitigated with advancing age. Even those aged >85 and also having renal insufficiency, hypotension or frailty, prescription of a BB and/or RAS inhibitor at discharge still had lower 1-year death or readmission rates.

**Worsening Renal Function.** Renal failure presents an obstacle and is a major challenge in managing HF patients. On the one hand, HF can compromise renal function due to decreased cardiac output and kidney perfusion, while HF medications may worsen renal function. The impact of HF medications on worsening renal function (WRF) and the link to outcome was examined in a post hoc analysis of TIME-CHF (NT-proBNP-guided vs. symptom-guided treatment in chronic HF) trial which comprised patients with LVEF ≤45% and ≥1 follow-up visits (*n* = 462) [117]. WRF III was defined as an elevation in serum creatinine ≥0.5 mg/dL at any time during the first 6 months. Patients with WRF III were prescribed on average increased loop diuretic doses (*p* = 0.0002) and higher spironolactone dose (*p* = 0.02), while the doses of beta blockers (*p* = 0.69) were similar and the doses of RAS blockers were lower (*p* = 0.09). There were important interactions between WRF III, medication and clinical outcome. Therefore, WRF III conferred poor prognosis if increased loop diuretic doses were administered (*p* = 0.001), but not with low doses (*p* = 0.29). The opposite was true for spironolactone (unfavorable prognosis in the case of WRF III without spironolactone, *p* < 0.0001; but not with spironolactone, *p* = 0.31). A protective action of beta blockers was confirmed in all patients (*p* < 0.001), but mostly in patients with WRF III (*p* < 0.05 for interaction). RAS blockers led to better outcome (*p* = 0.006), regardless of WRF III. The authors concluded that increased doses of loop diuretics might have deleterious effects, particularly when combined with significant WRF, whereas spironolactone and beta blockers might protect these patients.

With regard to diuretics having an adverse effect on kidney function, one needs to consider this in light of evidence indicating that when a diuretic is used as part of combined HF management, the prognosis is improved [118]. Indeed, it was reported that chronic use of loop diuretics was significantly linked with worsened prognosis in HF patients in a dose-dependent manner, while adding an RAAS inhibitor and a beta blocker conferred an improved prognosis, particularly when low-dose loop diuretics were employed in patients with low eGFR or LVEF [118]. Thus, the triad of the RAAS inhibitor, MRA and β-blocker improves prognosis when administered in combination with low-dose loop diuretics.

A recent investigation where data from two RCTs (PARADIGM-HF and PARAGON-HF) were reanalyzed dealt with the caveat of worsening renal function when transitioning from the RAAS inhibitor therapy to ARNi therapy [119]. Among patients randomly allocated to ARNi therapy, 11% in PARADIGM-HF and 10% in PARAGON-HF were noted to have an estimated glomerular filtration rate (eGFR) decrease (>15%) during the sacubitril/valsartan run-in phase. The eGFR was partly restored irrespective of the continuation of sacubitril/valsartan or changing therapy to an RAS inhibitor. Initial eGFR worsening was not consistently linked with clinical outcomes in either trial. Advantages of sacubitril/valsartan vs. RAS inhibitor therapy on clinical outcomes were not altered regardless of run-in eGFR drop in PARADIGM-HF (eGFR drop, HR: 0.69; 95% CI: 0.53–0.90; and no eGFR drop, HR: 0.80; 95% CI: 0.73–0.88; P_interaction_ = 0.32) and PARAGON-HF (eGFR drop, rate ratio, RR: 0.84; no eGFR drop, RR: 0.87; P_interaction_ = 0.92). The influence of sacubitril/valsartan therapy was still consistent across a range of eGFR decreases. The authors concluded that the long-term benefits in HF remain even after a moderate eGFR decrease is observed when transitioning from RASi to sacubitril/valsartan therapy and early eGFR fluctuations should not influence treatment with sacubitril/valsartan or avert up-titration.

**Neurohormonal Modulation in Heart Failure with Preserved Ejection Fraction. **Although HF with preserved ejection fraction (HFpEF) is a heterogeneous syndrome resulting from the interaction between various CVDs, comorbidities and ageing, this type of HF is also characterized by the activation of neurohormonal axes, particularly of the RAAS and the SNS, albeit to a lesser degree compared with HF with reduced ejection fraction (HFrEF) [75]. Importantly, RCTs have not shown a benefit from neurohormonal modulation treatments in HFpEF, perhaps with the exception of patients with LVEF in the lower normal range, whereby the American HF guidelines indicate that such treatment may be considered [61].

**Neurohormonal Blockade in End-Stage HF.** Finally, beta blocker and ACEi/ARB therapies have been applied and even conferred a reduction in HF recurrence and survival rate during the late period in patients with LV assist devices (LVAD) [120,121,122]. Specifically, a retrospective study of 64 patients on LVAD support of whom 31 received concurrent neurohormonal blockade therapy indicated that the combined endpoint of CV mortality or HF hospitalization was considerably decreased in patients receiving neurohormonal blockade therapy (*p* = 0.013) principally due to a 12.1% absolute decrease in HF-related hospitalizations (*p* = 0.046) attributed to a significant reversal in adverse myocardial remodeling and a decrease in morbidity and mortality versus LVAD support alone [120]. Also, a retrospective cohort analysis of the Interagency Registry for Mechanically Assisted Circulatory Support (INTERMACS) comprising 12,144 patients (21% women, 67% white, 25% African American and 6% Hispanic; mean age 57 ± 13) years), of whom 10,419 (85.8%) were receiving neurohormonal blockade (NHB), indicated that those having any NHB agent at 6 months had a better survival rate at 4 years versus patients not being treated with NHB (56% vs. 43.9%) [122]. After adjusted sensitivity analyses, this trend persisted with patients being on triple therapy with an angiotensin-converting enzyme inhibitor or angiotensin receptor blocker, β-blocker and mineralocorticoid antagonist demonstrating the lowest risk of death versus patients in the other subsets (hazard ratio, HR, 0.34). Compared with patients not taking NHB, the use of NHB was linked with a higher Kansas City Cardiomyopathy Questionnaire score (*p* = 0.02) and a 6 min walk test (*p* < 0.001).

**Neurohumoral Blockade in Cardiac Resynchronization Therapy.** Cardiac resynchronization therapy (CRT) has been shown to improve morbidity and mortality in patients with chronic HF on top of optimized medical treatment [123]. A study assessed the influence of HF medication optimization following CRT on morbidity and mortality in 185 HF patients after CRT implantation [124]. Over a mean follow-up of 44.6 months, 83 patients had a primary endpoint (death, heart transplantation, assist device implantation or hospitalization for HF). Therapy containing higher dosages of an ACEI or ARBs (*p* = 0.001) and beta blockers (*p* < 0.001) as well as therapy with lower doses of loop diuretics (*p* < 0.001) reduced the risk for the primary combined endpoint and for the all-cause death rate. Echocardiographic super-responders to CRT were able to receive higher mean dosages of ACEI/ARBs (68.1 vs. 52.4%, *p* < 0.01) and beta blockers (59 vs. 42.2%, *p* < 0.01). In the follow-up, the mean dose of loop diuretics was reduced by 20% in super-responders but raised by 30% in non-super-responders (*p* < 0.03). The authors concluded that, whenever possible, increasing neurohormonal blockade with the usage of higher dosages of neurohormonal blockers and lower dosages of diuretics had a beneficial effect with decreased morbidity and mortality after CRT implantation.

As mentioned, CRT has been shown to improve cardiac sympathetic nerve activity in responders and thus lead to reverse remodeling [58]. Similarly, long-term CRT was shown to reduce RAS activation and stabilize NT-proBNP in HF patients with reverse remodeling [59].

## 13. Salt Intake and Heart Failure/The Role of Neurohormonal Activity

Dietary salt consumption in patients with HF has been a controversial issue. Increased salt intake may cause arterial stiffness in susceptible individuals as it lowers nitric oxide (NO) release and increases endothelin-1 expression, overactivity of the renal SNS and also through aldosterone-independent activation of the mineralocorticoid receptor [125]. Salt restriction in these persons lowers arterial blood pressure (BP). Importantly, hypertension is an important factor for HF [126]. The optimal level of salt restriction that may improve CV outcomes remains debatable. Current guidelines suggest low sodium intake for the general population [127]. However, certain individuals do not develop arterial hypertension in response to sodium loading. Furthermore, data point to harmful effects of aggressive sodium restriction, even in HF patients [128]. Hence, the tendency currently is to recommend the consumption of a moderate range of dietary sodium (2.3–4.6 g/day; 1–2 teaspoons of salt) which is not linked with elevated CV risk, while the risk of CV disease seems to increase when sodium consumption exceeds 5 g/day [129]. A range of 3 to 5 g/d of sodium seems to be optimal for CV health. At this range, one attains BP advantages of lowering sodium consumption without the activation of RAAS, and the epidemiological data indicate the lowest rate of CV events [129].

Not only the high BP values are related to an elevated sodium consumption, but also arterial stiffness, oxidative stress and systemic inflammation seem to occur [130,131]. Elevated renin and aldosterone effects all promote cardiac hypertrophy and fibrosis, leading to HF [69]. The DASH and Mediterranean diets, which limit foods with high content in sodium, are helpful in the primary prevention of HF [132]. Sodium restriction is also suitable for the early stages of HF (“at risk” or “asymptomatic”). Finally, sodium restriction is considered to improve the outcomes of class III to IV HF by lowering BP measurements, aldosterone levels, the concentrations of B-type natriuretic peptide, plasma renin activity, cardiac filling pressure and oxidative stress [133]. However, it appears that moderate sodium restriction up to 2.8 g/day is even more useful in HF, versus low sodium restriction of up to 1.8 g/day [133]. Recent reviews indicate that a dietary sodium restriction of 2.6–3 g/day is effective in reducing the deleterious neurohormonal pathogenesis of HF [134]. This adds credence to pre-existing data demonstrating that low sodium restriction (<3 g/day) is linked with worse CV prognosis compared to moderate (3–5 g/d) sodium consumption [135]. Most evidence converges toward 2.6–3 g/day of dietary sodium as being efficacious for reduced BNP, renin and aldosterone plasma concentrations in patients with HF (neurohormonal homeostasis) [134]. For the general population, a J-shaped relationship between sodium consumption and CV events has been shown, based on trials from >300,000 persons, suggesting that the lowest risk of CV events and mortality is encountered in populations consuming what is considered an average range of sodium intake (3–5 g/d) [135].

## 14. Combination Therapies

Combined anticongestive therapies in patients with HF is common clinical practice. A systematic network meta-analysis of 75 clinical trials encompassing 95,444 individuals indicated that combined treatment with an ARNi, beta blocker, MRA and SGLT2i was most efficacious in lowering all-cause mortality (HR: 0.39); followed by an ARNi, beta blocker, MRA and vericiguat (HR: 0.41); and an ARNi, BB and MRA (HR: 0.44) [136]. The outcomes were similar for the composite endpoint of CV death or first hospitalization for HF (HR: 0.36 for ARNi, BB, MRA and SGLT2i; HR: 0.44 for ARNi, BB, MRA and omecamtiv mecarbil; and HR: 0.43 for ARNi, BB, MRA and vericiguat). The estimated extra number of life years gained for a 70-year-old patient on an ARNi, MRA, BB and SGLT2i was 5 years (range 2.5–7.5 years) versus no therapy in secondary analyses. Thus, the greatest benefit lies with a combination of an ARNi, BB, MRA and SGLT2i, whereby the deleterious influences of the two major systems of maladaptive neurohumoral activation (RAAS and SNS) are counteracted.

## 15. Natriuretic Peptides as Therapeutic Agents

As detailed above, ***natriuretic peptides***, brain (B-type) natriuretic peptide (BNP) and N-terminal prohormone of brain natriuretic peptide (NT-proBNP), are useful as biomarkers to confirm the presence and severity of HF [89,137]. However, these agents could also be potentially useful treatment modalities for HF patients. Nesiritide, a recombinant BNP, was granted approval by the US Federal Drug Administration (FDA) in 2001 for the management of acute HF based on its anticongestive effects comprising a decrease in pulmonary capillary wedge pressure and early relief of shortness of breath [50]. However, subsequently, several meta-analyses of the results of clinical trials with nesiritide raised concerns about a potential rise in the death rate and renal insufficiency conferred by this agent [138,139]. Also, the ASCEND-HF (Acute Study of Clinical Effectiveness of Nesiritide and Decompensated Heart Failure) trial was carried out to further examine the safety and efficacy of nesiritide in acute HF [140]. This trial randomly allocated 7141 patients with acute HF (irrespective of EF and within 24 h of intravenous therapy) to nesiritide or placebo for 24–168 h, with coprimary endpoints of an early (6–24 h) change in dyspnea and 30-day rehospitalization for HF or death. Self-reported dyspnea was marginally improved by nesiritide. There was no decrease in a 30-day rehospitalization rate for HF or death with nesiritide. Nesiritide was not linked with a worsening of renal function, but it was linked with higher rates of hypotension [140,141]. Other trials such as TRUE-AHF (a phase III, multicenter, randomized, double-blind, placebo-controlled trial to Evaluate the Efficacy and Safety of Ularitide (Urodilatin) Intravenous Infusion in Patients Suffering From Acute Decompensated Heart Failure), which explored the effects of the renal natriuretic peptide ularitide within 12 h of assessment, did not show clinical advantages [142]. Geographical variations in terms of participants’ characteristics, concomitant therapies and trial execution have also been reported in ASCEND-HF, which may have influenced trial results [143]. Thus, it remains a moot point whether short-term infusions might produce longer-term clinical benefits. Furthermore, the event rate was lower than expected in ASCEND-HF [140], such that much bigger trials may be necessary to demonstrate the clinical advantage of natriuretic peptide therapies for patients with acute HF. Finally, the question whether nesiritide is the optimal natriuretic peptide for HF management remains unanswered, considering its risk–benefit profile (with concerns related to hypotension). Additional considerations regarding this medication include whether or not a bolus is required, what the optimal dosing or duration of therapy might be, as well as whether or not follow-up infusions are necessary. Thus, ASCEND-HF and TRUE-AHF provide important insights concerning the therapeutic use of natriuretic peptides as well as insights relevant to designing and carrying out future trials. ANP has vasodilatory and natriuretic effects and inhibits the RAA system, and thus it can improve the LV function in HF patients [144,145]. In Japan, a recombinant human ANP, carperitide, has been employed for the management of acute HF since 1995; it has been reported that the outcomes in patients with acute HF are better, and the agent can decrease pulmonary congestion in patients with systolic blood pressure of ≥120 mmHg [146]. However, in a recent (2019) report from the Japanese registry database, authors reported that carperitide conferred worse outcomes when compared to nitrates [147]. In contrast, the consortium for pooled data analysis regarding hospitalized patients with HF in Japan, the COOPERATE-HF-J (Consortium for Pooled Data Analysis regarding Hospitalized Patients with Heart Failure in Japan) study, disclosed that low-dose carperitide ameliorated the prognosis of patients with acute HF within one year after admission [148]. The findings of this trial suggest that blood pressure and renal function may impact the efficacy of carperitide [149]. In order to clarify the prognostic influence of carperitide in HF, large, well-designed clinical trials, especially focusing on its dosage, are certainly needed.

Several relevant issues need to be addressed when attempting to use NPs for HF treatment [150]. Unfortunately, to date this strategy has not been demonstrated to ameliorate clinical outcomes in sufficiently powered trials. Although nesiritide (recombinant human BNP) was approved for the management of acute decompensated HF, the following ASCEND-HF (A Study Testing the Effectiveness of Nesiritide in Patients with Acute Decompensated Heart Failure) trial did not show any clinical advantages [140]. A smaller trial from the National Heart, Lung, and Blood Institute Heart Failure Network (ROSE (Renal Optimization Strategies Evaluation) trial) using low doses of nesiritide did not show clinical improvement in hospitalized patients with acute HF [151]. Also, long-term treatment with intermittent infusion of nesiritide (FUSION-2 (Second Follow-Up Serial Infusions of Nesiritide)) in high-risk patients was neutral [152]. In addition, all three studies indicated a higher risk of hypotension conferred by this agent. On the other hand, a strategy worth investigating relates to a low-dose, long-term therapy via subcutaneous approach that may minimize the risk of hypotension observed with intravenous therapy; also, use in earlier stage HF patients could be more efficacious than in patients with advanced HF included in the aforementioned trials.

## 16. Cardiac Arrhythmias and Sudden Cardiac Death/Arrhythmogenesis in Heart Failure

Cardiac arrhythmias in HF patients may relate to the imbalance of the autonomic nervous system (ANS) in these patients where there is preponderance of the SNS activity accounting for both atrial and ventricular arrhythmias [4,10,153,154]. Intricate mechanisms, varying for specific cardiac arrhythmias, are implicated in this arrhythmogenic process. A plethora of signaling pathways have been shown to be responsible for these (maladaptive) remodeling processes and arrhythmogenic events in HF patients, comprising the beta adrenergic pathway, RAAS and Ca-calmodulin-dependent kinase II and calcineurin-mediated signaling [4]. The “external” triggers of arrhythmias such as mechanical stretch (volume overload), neurohumoral activation or other stressors (e.g., systemic inflammation) are also worth mentioning [4].

There are more data available for the arrhythmogenic effects of the SNS, which, when overactive, can trigger atrial and/or ventricular “adrenergic” arrhythmias in susceptible individuals (e.g., ischemic heart disease or cardiomyopathies), while it can also reverse or negate the protective effects of the antiarrhythmic drugs [10,155]. However, there is also evidence that parasympathetic overactivity may trigger “vagotonic” arrhythmias (e.g., paroxysmal AF, Brugada syndrome, idiopathic ventricular fibrillation). Thus, a fine balance is necessary to be attained between these two limbs (sympathetic/parasympathetic) of the ANS in order to preserve eurhythmia, which is a hard task to accomplish and sustain [10]. In many arrhythmogenic diseases, including the syndrome of HF, ANS modulation remains an investigative tool [156].

Sudden cardiac death (SCD) accounts for up to 50% of deaths in patients with HF (attributable to malignant ventricular arrhythmias, proportionally more often in patients with HFrEF vs. HFpEF) [154], depending on the severity of symptomatic functional impairment (NYHA class) and LV dysfunction [157]. Its share is increased in the early stages of HF. Countering neurohormonal activation with therapies directed at the RAAS and ANS may lower the SCD rates via improved hemodynamics, decreased SNS activity in the myocardium and the inhibition of cardiac remodeling [158,159,160,161,162]. The angiotensin-converting enzyme (ACE) inhibitors decrease the overall death rate in chronic HF, and the greatest benefit seems to arise from delayed HF progression rather than reduced SCD. In HF patients who experience myocardial infarction (MI), a lower incidence in SCD may contribute to a greater degree to the mortality benefits of ACE inhibition. Added beta blocker therapy to ACEi/ARBs has consistently curtailed the SCD rate by one-third vs. placebo in patients with either mild-to-moderate or severe HF and in the presence or absence of MI. Aldosterone blockade when added to an ACEi reduces the risk of SCD in advanced chronic HF and when given in addition to both ACEi and beta blocker in HF linked with acute MI [10]. For maximal SCD risk reduction in HF, beta blocker therapy should be combined with standard ACE inhibitor therapy, while aldosterone blockade may be added for particular groups of HF patients [4].

As detailed herein, HF is associated with neurohormonal activation that includes sustained elevations in SNS and RAAS signaling, which lengthen the action potential duration (APD) and predispose to arrhythmogenic SCD via pathologic control of ion channels, including I_Ks_ [163,164]. In HF patients, there is pathologic regulation and remodeling that alters I_Ks_ with its attendant clinical implications on electrical instability and arrhythmogenesis [165]. Importantly, the RAAS is a critical component of the electrical remodeling of the failing heart, and the RAAS blockade lowers the risk of SCD, with the ARBs representing a robust tool to enhance overall survival and possibly curtail the risk of SCD provided that adequate drug doses are used to reach optimal AT1-receptor blockade [162]. Importantly, the efficacy of sacubitril/valsartan, a combined RAAS blocker, was assessed in patients with HFrEF carrying an implantable cardiac defibrillator (ICD) or a cardiac resynchronization therapy with a defibrillator (CRT-D); this combined drug therapy seemed to decrease the risk of arrhythmic events requiring appropriate shock therapy.

Furthermore, HF is also known to pathologically regulate a broad spectrum of ion channels and calcium-handling proteins, which contribute to HF-linked ventricular arrhythmogenesis. Prior evidence has pointed to pathological regulation of ventricular sodium (e.g., late I_Na_), calcium (e.g., ICa,L) and potassium currents (e.g., the transient outward potassium current, IKr, the inward rectifier potassium current) along with calcium-handling proteins, such as the ryanodine receptor, the sodium–calcium exchanger and sarcoplasmic/endoplasmic reticulum Ca_2+_-ATPase, in HF [165,166,167,168,169]. These HF-associated changes in affected ionic currents with resultant deficits in other repolarizing ionic currents, including I_Kr_ and the inward rectifier potassium current, the arrhythmogenic potential of reductions in I_Ks_ is amplified in HF [165,170,171]. Indeed, it has been shown that HF-linked reductions in I_Ks_ contribute to the proarrhythmic effects of other calcium-related ion carriers that are activated in response to β-AR stimulation (i.e., I_Ca,L_, the sodium–calcium exchanger) [172,173].

Sympathetic overactivity may amplify the expression of Na_+_-Ca_2+_ exchanger in end-stage HF; current data lend support to the hypothesis that enhanced Na_+_-Ca_2+_-exchange could promote malignant ventricular arrhythmias [169]. Thus, the ventricular electrical instability and arrhythmogenesis that characterize HF emanate from complex interaction of multiple ionic currents that are subjected to pathological remodeling in HF.

Although the prediction of SCD in HF remains an unmet need, recent prospective cohort data indicate that the proportion of SCD in outpatients with HF seems to be lower than expected; whether this is the result of the implementation of guideline-directed therapies in HF remains to be shown [174].

With regard to the impact of countering the RAAS on arrhythmias in HF, a recent meta-analysis of ten trials, including six RCTs and four observational studies (18,548 patients with 9328 patients in the ARNI group and 9220 patients in the ACEI)/ARB group) indicated that over a median of 15 months, a considerable decrease was noted in the composite outcomes of SCD and ventricular arrhythmias in patients receiving ARNIs vs. those on ACEIs/ARBs (odds ratio, OR 0.71; *p* = 0.01; I^2^ = 17%) [175]. ARNI therapy also led to a significant decrease in ICD shocks. The incidence rate of specific arrhythmias (VT, VF, AF or SVT) did not differ in the two groups. The authors concluded that the use of ARNIs reduced the composite outcomes of SCD and ventricular arrhythmias in patients with HF; these results were mainly driven by a decrease in SCD in patients managed with ARNIs.

With regard to the effect of SGLT2 inhibitors on SCD, the data are promising. The primary pharmacological action of SGLT2 inhibitors is to block the reabsorption of glucose and sodium ions from the proximal tubules of the kidney and to facilitate urinary glucose excretion [9]. Many clinical studies have provided robust data on their potent protective effects in patients with HF or chronic kidney disease (CKD), irrespective of the presence or absence of diabetes. However, the data on the effect of SGLT2 inhibitors on SCD or fatal ventricular arrhythmias (VAs) are only slowly accumulating [176]. Several cardiorenal protective actions of SGLT2 inhibitors result in hemodynamic improvement, reverse remodeling of the failing heart, decreased sympathetic hyperactivity, correction of electrolyte disturbances, restoration of anemia and iron metabolism, antioxidative and antifibrotic effects, which may all contribute to the prevention of SCD and/or VAs [107]. Besides indirect cardioprotective effects, SGLT2 inhibitors may also have possible direct myocardial effects not only via the inhibition of Na^+^/H^+^ exchanger (NHE) activity but also via the suppression of aberrantly enhanced late Na^+^ current and thus may participate in the prevention of SCD and/or VAs via the restoration of the lengthened repolarization phase in the failing myocardium. In keeping with their promising and favorable hemodynamic effects, a recent meta-analysis of 11 trials reporting on SCD in patients receiving SGLT2 inhibitors, indicated that these agents seem to have a favorable effect on SCD [177]. SCD was reported in seven of the eleven trials meeting selection criteria; 10,796 patients were placed on SGLT2i, and 10,796 on placebo. SGLT2i treatment conferred a significant reduction in the risk of SCD (risk ratio, RR: 0.68; *p* = 0.003; I^2^ = 0%). Nevertheless, further data will be needed from prospective trials in order to establish the long-term influence of SGLT2i agents on ventricular arrhythmias.

While aiming at the SNS and RAAS signaling in HF has beneficial effects [178,179], novel potential therapeutic targets are being disclosed which are encountered downstream of membrane-bound receptors that have the potential to surpass challenges associated with signaling pathway crosstalk [180,181]. Potential advantages of these promising new therapeutic goals may be, at least in part, ascribable to their ability to avert or improve pathological I_Ks_ remodeling. Nevertheless, more research is required to examine the therapeutic potential of these new targets with regard to arrhythmogenic SCD, besides offering other avenues in HF therapies. The next therapeutic paradigm may comprise an integration of current and newer hemodynamic modulators with more patient-specific targeting of maladaptive molecular pathways.

**Patients with HFpEF**. In patients with HF with preserved ejection fraction (HFpEF), SCD is responsible for about 25–30% of the all-cause death rate and 40% of the CV death rate [182]. The mechanism of SCD in HFpEF remains unknown, but arrhythmic events are deemed to predominate. There are only a few data in the literature indicating a higher occurrence of VAs in patients with HFpEF vs. the general population. Increased rates of clinically important conduction system disease and the need for cardiac pacing are encountered in patients with HFpEF vs. the general population. Nevertheless, the occurrence of SCD in the HFpEF population remains high, and there would be a dire need for therapeutic interventions if instigated by VAs.

**Device Therapy**. Although several editorials allege that guideline-directed therapy, of which the major part involves agents that counteract neurohumoral activation in HF patients, the risk of SCD remains crucial and despite a relative SCD risk reduction noted in HF trials, the residual risk is still substantial (of course, this is also commensurate with patient compliance to drug therapy), which justifies the need for device therapy, either as an implantable cardioverter defibrillator (ICD) or cardiac resynchronization therapy (CRT) defibrillator (CRT-D) [123,183]. Indeed, over the past decade, survival has improved for patients with HF both as a result of drug and device therapies, despite an increasing comorbidity burden [184].

## 17. Updated Guideline-Directed Therapies for HF

According to the latest (2021) European guidelines [60], ACEi or ARNi, beta blockers, MRAs and SGLT2 inhibitors are advised as principal therapies for patients with HFrEF. ACEi/ARNi, beta blockers and MRAs may be considered in patients with HF with mid-range LVEF (HfmrEF). They also state that to date, no therapy has been demonstrated to decrease mortality and morbidity in patients with HFpEF.

Per the latest (2022) American guidelines, guideline-directed medical therapy (GDMT) for HF with reduced ejection fraction (HFrEF) should now comprise four medication categories including sodium-glucose cotransporter-2 inhibitors (SGLT2i) [185]. These four classes are (1) renin–angiotensin system inhibition with angiotensin receptor–neprilysin inhibitors (ARNi), angiotensin-converting enzyme inhibitors (ACEi) or angiotensin (II) receptor blockers (ARBs) alone; (2) beta blockers; (3) mineralocorticoid receptor antagonists (MRAs); (4) the new group, SGLT2i. The SGLT2 inhibitors have a class of recommendation 2a in HF with mildly reduced or mid-range ejection fraction (HFmrEF). Weaker recommendations (class 2b) are put forth for ARNi, ACEi, ARBs, MRAs and beta blockers in this population. Also, new recommendations for HFpEF are issued for SGLT2i (class 2a), MRAs (class 2b) and ARNi (class 2b). Several previous recommendations have been renewed and these include treatment of hypertension (class 1), management of atrial fibrillation (class 2a), usage of ARBs (class 2b) and precluding the routine use of nitrates or phosphodiesterase-5 inhibitors (class 3: no benefit).

## 18. Conclusions

The neurohormonal activation is now recognized as one of the most important mechanisms underlying the progression of HF, and the therapeutic antagonism of the involved neurohormonal systems has become the cornerstone of contemporary pharmacotherapy for HF. In this regard, all three neurohumoral systems (sympathetic system, RAAS and AVP system) seem to have a significant, albeit variable, role and impact in HF progression, prognosis and outcome (Figure 1, Table 1). Specific biomarkers of the activation of these neurohormonal pathways, such as the natriuretic peptides, catecholamine levels and neprilysin and various newer ones, may be employed to better elucidate and gain insight into the mechanisms of HF drugs and also help to identify the subgroups of patients who might derive a benefit from specific therapies, irrespective of the degree of LV dysfunction. Future studies need to determine whether certain patients or patient groups respond better to specific guided therapies targeting individual components of the neurohormonal systems rather than to blind combination therapies.

## 19. Perspective

Although the activation of these neurohormonal systems preserves CV homeostasis in the beginning of HF and for the short-term, a plethora of studies, both pre-clinical and clinical trials, have demonstrated that the bioactive molecules generated by the activation of these three systems (catecholamines, angiotensin II, aldosterone and AVP) are becoming toxic to the heart at higher levels. Thus, counteracting and blocking these molecules by respective agents such as the β-blockers, ACE inhibitors, ARBs, aldosterone antagonists and vaptans, and more recently with the use of ARNIs, can negate the deleterious consequences of such neurohormonal activation. The recent use of the novel ARNI drug class has led to superior efficacy of multiple-acting neurohormonal blockade in chronic HF. Some of these agents also restore the imbalance between endogenous vasoconstrictors (such as angiotensin II) and vasodilators (such as the natriuretic peptides) that emanate from sustained neurohormonal activation. However, although neurohormonal antagonists aid in the stabilization of the symptomatology of HF and enhance reverse LV remodeling, the “remission” from HF is self-limiting and symptoms eventually recur. Thus, although the hypothesis of the neurohormonal activation has greatly expanded our understanding of the HF syndrome, this model does not fully explain and justify the disease progression. Further studies focusing directly on individual biological mechanisms and components of the neurohormonal axes participating in LV remodeling might furnish new insights into the pathophysiology of HF progression and usher in and guide the development of newer remedies. Finally, patients with HFpEF and HF with concomitant AF represent major remaining clinical challenges that seem to be less responsive to current pharmacotherapy. Novel neurohormonal blocking agents and the refined use of current drugs, as well as up-titration to recommended target doses, are direly needed to curtail the adverse clinical events and to ameliorate the outcomes in HF.

## Figures and Tables

**Table 1:** Neurohormonal activation in heart failure (HF): consequences/current developments/future directions.

**I. Activation of the Sympathetic Nervous System (SNS)**
Occurs early in the course of the disease/leads to increased circulating levels of norepinephrine (NE) secondary to increased SNS signaling and NE release from adrenergic nerves and attendant “spillover” into the plasma and also from reduced uptake by adrenergic nerve endings/High NE levels correlate directly with the severity of HF and inversely with survival; Sustained activation of the SNS exerts deleterious effects on the heart, kidneys and peripheral vasculature, such as desensitization of the beta adrenergic receptors, myocyte hypertrophy/necrosis/apoptosis, myocardial fibrosis, renal arterial and venous vasoconstriction, activation of RAAS, salt and water retention, attenuated response to NPs, peripheral vasoconstriction and vascular hypertrophy and cardiac arrhythmias; Counteracted by beta blockers/also attenuated by effective treatment of HF with other agents, e.g., ACEIs.
II. Activation of the Renin–Angiotensin–Aldosterone System (RAAS) * †
Vasoconstriction; Increased salt and water retention; RAAS–counterregulatory hormone release (ANP, BNP); Provoked release of AVP; Counteracted by ACEIs, ARBs, MRAs and dual-acting angiotensin receptor–neprilysin inhibitors (ARNIs).
**III. Activation of the Arginine Vasopressin (AVP) System**
Water retention/hyponatremia; Increased peripheral vasoconstriction/endothelin production; Counteracted by vasopressin receptor antagonists (vaptans).
**IV. Natriuretic Peptides (NPs)**
Antagonizing angiotensin II; Diuretic/natriuretic actions; Vasodilatory actions; Inhibition of aldosterone secretion; Sensitive markers of cardiac load (biomarkers); However, a therapeutic strategy with the use of NPs has not been demonstrated to ameliorate clinical outcomes in sufficiently powered clinical trials.
**V. Sodium-Glucose Co-Transporter-2 (SGLT2) Inhibitors**
SGLT2 inhibitors could be viewed as neurohormonal antagonists when prescribed for the management of HFrEF; Their benefits extend beyond glycemic control, since the inhibition of renal glucose reabsorption has an effect on blood pressure and ameliorates the hemodynamic profile and the tubule glomerular feedback; such an effect can rebalance the dense macula response by restoring adenosine formation and limiting RAAS activation; Furthermore, there is also evidence supporting the notion of potential sympatholysis exerted by SGLT2 inhibitors independently of their glucose-lowering and diuretic effects; Finally, antiarrhythmic actions have been ascribed to SGLT2 inhibitors via various mechanisms (see text for discussion).
**VI. Future Directions**
The recent development of the novel ARNI drug class and also the introduction of SGLT2 inhibitors in HF therapy has led to enhanced efficacy of multiple-acting neurohormonal blockade in chronic HF; The effect seems to be most beneficial when medications from the four main drug classes (sartans, beta blockers, MRAs and SGLT2 inhibitors) are used in conjunction in HF patients; However, we are in dire need of studies that will assess the safety and efficacy of a gradual sequential initiation or an accelerated addition of HF drugs and up-titration of doses as long as a hypotensive effect is avoided, and such a polypharmacy approach is well tolerated; Future studies of neurohormonal activity suppression in HF patients need to assess not only symptom relief and quality of life but also the hard endpoints of total, CV and HF mortality; Finally, gene and stem cell-based therapies for HF patients may turn out to be promising future treatment modalities that might also affect the neurohumoral balance.

ACEI(s) = angiotensin-converting enzyme inhibitor(s); ANP = atrial natriuretic peptide; ARB = angiotensin receptor blocker; ARNIs = angiotensin receptor–neprilysin inhibitors; AVP = arginine vasopressin; BNP = brain natriuretic peptide; CV = cardiovascular; HFrEF = heart failure with reduced ejection fraction; MRA = mineralocorticoid receptor antagonist; NE = norepinephrine; NP = natriuretic peptide; RAAS = renin–angiotensin–aldosterone system; SGLT2 = sodium-glucose co-transporter-2; SNS = sympathetic nervous system. * Additional short-chain peptides from angiotensin II, including heptapeptide angiotensin III and the hexapeptides angiotensin IV and angiotensin 1–7, serve as effectors in the RAAS. † despite the use of ACE inhibitors and ARBs, aldosterone levels increase in ~30–40% patients with HF (phenomenon of “*aldosterone escape*” or “*aldosterone breakthrough*”, thought to be secondary to an increased level of renin, although the exact pathophysiology remains unknown).

## Data Availability

No new data were created or analyzed in this study. Data sharing is not applicable to this article.

## Abbreviations

ACE = angiotensin-converting enzyme; ARNi = angiotensin receptor–neprilysin inhibitors; AVP = arginine vasopressin; BNP = brain natriuretic peptide; CI = chronotropic incompetence; eGFR = estimated glomerular filtration rate; HF = heart failure; HR = heart rate; MRAs = mineralocorticoid receptor antagonists; RAAS = renin–angiotensin–aldosterone system; SGLT2i = sodium-glucose co-transporter-2 inhibitors; SNS = sympathetic nervous system; WRF = worsening renal function.
